# Additive prognostic value of red cell distribution width over late gadolinium enhancement on CMR in patients with non-ischemic dilated cardiomyopathy

**DOI:** 10.1038/s41598-020-66198-0

**Published:** 2020-06-08

**Authors:** Jeong-Eun Yi, Hye-Jeong Lee, Young Jin Kim, Yookyung Kim, Boyoung Joung, Junbeom Park

**Affiliations:** 1 Department of Cardiology, Eunpyeong St. Mary’s Hospital, The Catholic University of Korea, Seoul, Republic of Korea; 2 Department of Radiology, Research Institute of Radiological Science, The Yonsei University College of Medicine, Seoul, Republic of Korea; 3 Department of Radiology, Ewha Womans Mokdong’s Hospital, Ewha Womans University College of Medicine, Seoul, Republic of Korea; 4 Yonsei University Health System, Yonsei Cardiovascular Hospital, Yonsei University College of Medicine, Seoul, Republic of Korea; 5 Department of Cardiology, Ewha Womans Mokdong’s Hospital, Ewha Womans University College of Medicine, Seoul, Republic of Korea

**Keywords:** Heart failure, Cardiac hypertrophy

## Abstract

Elevated red cell distribution width (RDW) and late gadolinium enhancement on cardiac magnetic resonance (LGE-CMR) are both poor prognostic factors. This study examined the relationship between RDW and LGE-CMR characteristics in patients with non-ischemic dilated cardiomyopathy (NICM), and investigated whether the additive prognostic value of RDW as an integrative systemic factor over LGE-CMR exists or not. A total of consecutive 378 patients who underwent CMR at two general hospitals in South Korea were retrospectively analyzed. The primary endpoint was a composite of all-cause death, hospitalizations due to worsening heart failure and major arrhythmic events. During a mean follow-up period of 40.8 months, 151 (39.9%) patients experienced primary endpoints. The RDW value was significantly higher in patients with LGE than in those without LGE (13.7 ± 1.5% vs. 13.3 ± 1.4%, p = 0.034), but it was not associated with the extent or distribution patterns of the LGE. Addition of RDW into the model with clinical risk factors and LGE-CMR characteristics led to a significant improvement in the prediction of worse outcomes (χ^2^ increased from 73 to 82; p = 0.023). RDW could provide incremental predictive value for adverse clinical events beyond LGE-CMR data in NICM patients.

## Introduction

Red cell distribution width (RDW) is a hematological parameter reflecting the degree of variation in the size of circulating erythrocytes and is used to differentiate the etiology of anemia^1^. Recently, the RDW has emerged as a poor prognostic marker among patients with various cardiovascular diseases, including coronary artery disease (CAD), peripheral artery disease (PAD), atrial fibrillation (AF), and heart failure (HF)^2^. Numerous studies have reported that an elevated RDW not only is a strong independent predictor of adverse outcomes in patients with HF^3^, but also predicts the risk of developing HF in the general population^4^. However, the exact mechanism linking an elevated RDW with a poor prognosis, particularly in patients with HF, is poorly understood^5^. To the best of our knowledge, there is a paucity of data on the clinical significance of the RDW in the risk stratification of non-ischemic dilated cardiomyopathy (NICM) for adverse cardiac events.

Cardiac magnetic resonance (CMR) imaging with gadolinium is a non-invasive imaging modality to accurately identify and quantify myocardial fibrosis known as a well-established risk factor of adverse cardiac events in patients with NICM^6^. Previous studies have demonstrated that the presence, extent, and distribution pattern of late gadolinium enhancement on CMR (LGE-CMR) can provide incremental prognostic information beyond the left ventricular ejection fraction (LVEF) in this population^6,7^. In some reports, although the presence of LGE had localized effects, it was associated with chronic inflammation that has been implicated in the pathogenesis of fibrosis^8–10^. However, LGE itself does not always mean the pathologic fibrosis or scarring of the myocardium^11^, and it is also unknown whether the progression and increase in the LGE could exactly reflect the underlying systemic inflammation. Moreover, the assessment of the LGE-CMR has limited ability to detect diffuse interstitial fibrosis, which is another subtype of myocardial fibrosis commonly found in patients with NICM^12^.

This study aimed to examine the association between RDW and LGE-CMR in patients with NICM, and further assessed the incremental value of RDW as a systemic marker beyond the localized LGE-CMR in the prediction of worse outcomes.

## Results

### Baseline characteristics

A total of 378 patients were included in the analysis, and the mean age was 55 ± 15 years and 237 (62.7%) were males. During a mean follow-up of 40.8 ± 36.1 months, the primary endpoint occurred in 151 (39.9%) patients. Among them, there were 112 (29.6%) of death, 61 (16.1%) of worsening HF, and 51 (13.5%) of major arrhythmic events. The baseline clinical characteristics are summarized in Table 1. Compared to the patients without events, subjects with events tended to be older and current smokers, and had a significantly lower body mass index (BMI) and diastolic blood pressure (BP) level. Patients with events had a significantly higher RDW level (13.9 ± 1.5% vs. 13.4 ± 1.4%, p = 0.001), along with lower hemoglobin, hematocrit, platelet (PLT) count, estimated glomerular filtration rate calculated using the Modification of Diet in Renal Disease formula (eGFR_MDRD_)_,_ total cholesterol (TC), and low-density lipoprotein cholesterol (LDL) levels than those without events. Although the proportion of diabetes mellitus (DM) was higher in the subjects with events than in those without events, the hemoglobin A1c level was similar between them. There were no significant differences in the other co-morbidities and N-terminal pro-brain natriuretic peptide (NT-proBNP) and C-reactive protein (CRP) levels between the two groups.

**Table 1:** Baseline characteristics.

Variables	Total (n = 378)	Death, Worsening HF, Major arrhythmic events		
		With events (n = 151)	Without events (n = 227)	p Value^*^
Age (years)	55 ± 15	56 ± 15	54 ± 15	0.056
Male, n (%)	237 (62.7)	94 (62.3)	143 (63.0)	0.884
Body mass index (kg/m^2^)	24.7 ± 4.4	24.1 ± 4.3	25.2 ± 4.5	0.034
Systolic BP (mmHg)	119 ± 19	117 ± 19	120 ± 19	0.239
Diastolic BP (mmHg)	75 ± 13	73 ± 13	76 ± 14	0.016
Heart rate (bpm)	81 ± 16	81 ± 15	81 ± 17	0.830
NYHA class ≥ 3, n (%)	195 (51.6)	78 (51.7)	117 (51.5)	0.983
Current smoker, n (%)	96 (25.5)	46 (30.7)	50 (22.0)	0.059
Diabetes mellitus, n (%)	107 (28.3)	54 (35.8)	53 (23.3)	0.009
Hypertension, n (%)	172 (45.5)	73 (48.3)	99 (43.6)	0.366
AF or AFL, n (%)	78 (20.6)	26 (19.2)	49 (21.6)	0.575
QRS duration (msec)	109 ± 25	111 ± 25	108 ± 25	0.445
QTc duration (msec)	467 ± 41	469 ± 44	466 ± 39	0.538
Medications				
ACEi or ARB, n (%)	349 (92.3)	137 (90.7)	212 (93.4)	0.341
Beta-blocker, n (%)	284 (75.1)	102 (67.5)	182 (80.2)	0.005
Loop diuretics, n (%)	210 (82.0)	130 (86.1)	180 (79.3)	0.092
Spironolactone, n (%)	246 (65.1)	109 (72.2)	137 (60.4)	0.018
Digoxin, n (%)	126 (33.4)	71 (47.0)	55 (24.3)	<0.0001
Amiodarone, n (%)	19 (5.0)	12 (7.9)	7 (3.1)	0.034
Antiplatelet, n (%)	180 (47.6)	76 (50.3)	104 (45.8)	0.389
Anticoagulant, n (%)	95 (25.1)	37 (24.5)	58 (25.6)	0.818
Statin, n (%)	122 (32.3)	52 (34.4)	70 (30.8)	0.463
WBC count (x10^3^/µL)	6930 (5740–8180)	6630 (5510–8000)	7100 (5900–8220)	0.160
Hemoglobin (g/dL)	13.8 ± 2.1	13.3 ± 2.1	14.2 ± 2.0	<0.0001
Hematocrit (%)	41.0 ± 6.0	39.4 ± 6.2	42.1 ± 5.6	<0.0001
Mean corpuscular volume (fL)	90.2 ± 5.1	89.7 ± 4.9	90.4 ± 5.2	0.684
Red cell distribution width (%)	13.6 ± 1.5	13.9 ± 1.5	13.4 ± 1.4	0.001
PLT count (x10^3^/L)	235 (186–290)	217 (175–273)	241 (198–294)	0.004
Fasting glucose (mg/dL)	113 ± 41	112 ± 32	113 ± 47	0.751
HbA1c (%)	6.6 ± 1.4	6.7 ± 1.3	6.5 ± 1.5	0.496
Blood urea nitrogen (mg/dL)	16.1 (13.0–21.5)	17.7 (13.4–24.2)	15.4 (13.0–20.1)	0.004
Creatinine (mg/dL)	1.0 (0.8–1.2)	1.0 (0.9–1.3)	1.0 (0.8–1.2)	0.008
eGFR_MDRD_ (mL/min/1.73m^2^)	76.0 (63.1–91.3)	72.6 (56.7–89.5)	78.0 (66.1–92.8)	0.004
Total cholesterol (mg/dL)	169 ± 38	161 ± 38	174 ± 39	0.002
Triglyceride (mg/dL)	123 ± 73	122 ± 78	124 ± 69	0.887
HDL cholesterol (mg/dL)	43 ± 14	42 ± 15	44 ± 14	0.157
LDL cholesterol (mg/dL)	107 ± 32	101 ± 29	110 ± 33	0.013
NT-proBNP (pg/mL)	2620 (1008–5746)	2952 (1102–6764)	2521 (862–5010)	0.212
C-reactive protein (mg/dL)	2.2 ± 2.4	2.3 ± 2.3	2.2 ± 2.5	0.755

*Comparisons between patients with and without events. *HF*, heart failure; *BP*, blood pressure; *NYHA*, New York Heart Association; *AF*, atrial fibrillation; *AFL*, atrial flutter; *ACEi*, angiotensin converting enzyme inhibitor; *ARB*, angiotensin II receptor blocker; *WBC*, white blood cell; *PLT*, platelet; *HbA1c*, hemoglobin A1c; *eGFR*_*MDRD*_, estimated glomerular filtration rate calculated using the Modification of Diet in Renal Disease formula; *HDL*, high-density lipoprotein; *LDL*, low-density lipoprotein; *NT-proBNP*, N-terminal pro-brain natriuretic peptide.

Table 2 shows LGE-CMR characteristics. The mean LVEF was 24.1 ± 8.9%. Patients with events had greater biventricular end-diastolic and end-systolic volume indices compared to those without events, however, the LV and right ventricular (RV) EF were similar between the two groups. LGE was present in 258 (68.3%) patients and the mean LGE extent was 7.3 ± 11.8%. LGE was more frequently observed in patients with events compared to those without events (82.8% vs. 58.6%, p < 0.0001), and the extent of the LGE was greater in patients with events (11.4 ± 14.8% vs. 4.5 ± 8.2%, p < 0.0001). The LGE distribution patterns were identified as midwall in 166 (43.9%), patchy in 123 (32.5%), transmural in 29 (7.7%), subendocardial in 16 (4.2%), and subepicardial in 9 (2.4%) patients, respectively. Midwall (55.6% vs. 36.1%, p < 0.0001), subepicardial (4.6% vs. 0.9%, p = 0.033), and transmural (11.9% vs. 4.8%, p = 0.011) patterns were more frequently observed in patients with events.

**Table 2:** CMR characteristics.

Variables	Total (n = 378)	Death, Worsening of HF, Major arrhythmic events		p Value^*^
		With events (n = 151)	Without events (n = 227)	
LVEF (%)	24.1 ± 8.9	23.8 ± 8.8	24.7 ± 9.0	0.166
LVEDVI (ml/m^2^)	154.7 (128.3–193.2)	166.5 (133.7–202.6)	143.9 (117.4–180.2)	0.001
LVESVI (ml/m^2^)	127.3 ± 54.5	140.2 ± 60.1	117.0 ± 47.4	0.001
RVEF (%)	33.7 ± 15.6	33.6 ± 15.4	33.7 ± 15.7	0.941
RVEDVI (ml/m^2^)	98.9 ± 41.4	108.3 ± 48.5	91.9 ± 33.9	0.007
RVESVI (ml/m^2^)	68.8 ± 38.0	75.6 ± 45.4	63.8 ± 30.8	0.035
Presence of LGE	258 (68.3)	125 (82.8)	133 (58.6)	<0.0001
Extent of LGE (%)	7.3 ± 11.8	11.4 ± 14.8	4.5 ± 8.2	<0.0001
Patterns of LGE				
Subendocardial, n (%)	16 (4.2)	8 (5.3)	8 (2.5)	0.401
Midwall, n (%)	166 (43.9)	84 (55.6)	82 (36.1)	<0.0001
Subepicardial, n (%)	9 (2.4)	7 (4.6)	2 (0.9)	0.033
Transmural, n (%)	29 (7.7)	18 (11.9)	11 (4.8)	0.011
Patchy, n (%)	123 (32.5)	54 (35.8)	69 (30.4)	0.276

*Comparisons between patients with and without events. *CMR*, cardiac magnetic resonance; *HF*, heart failure; *LVEF*, left ventricular ejection fraction; *LVEDVI*, left ventricular end diastolic volume index; *LVESVI*, left ventricular end systolic volume index; *RVEF*, right ventricular ejection fraction; *RVEDVI*, right ventricular end diastolic volume index; *RVESVI*, right ventricular end systolic volume index; *LGE*, late gadolinium enhancement.

### Relationship between RDW and LGE-CMR characteristics

**Figure 1:**
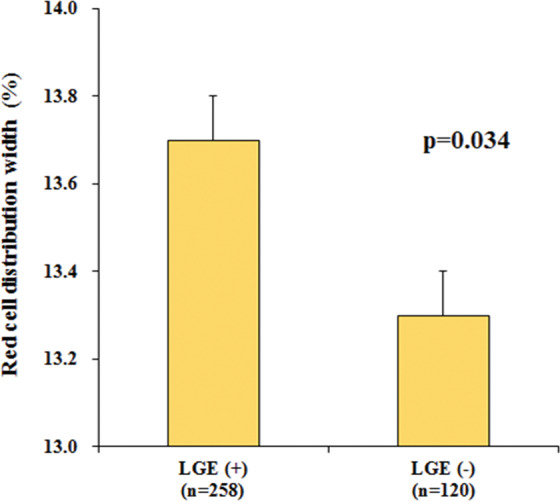
Comparison of the mean level of RDW between the patients with and without LGE *RDW*, red cell distribution width; *LGE*, late gadolinium enhancement.

Patients with LGE had a higher RDW level than those without LGE (13.7 ± 1.5% vs. 13.3 ± 1.4%, p = 0.034) (Fig. 1). However, RDW was not correlated with the extent of LGE (p = 0.779) and there were no significant differences in the RDW level across the distribution pattern of LGE (p = 0.164).

### Clinical outcomes

**Figure 2:**
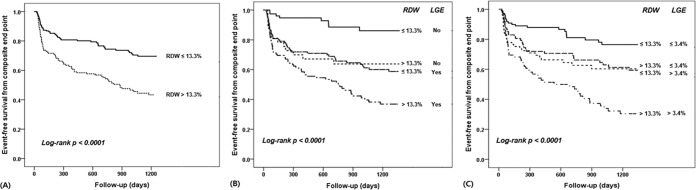
Kaplan-Meier survival curves in the groups of patients categorized by the RDW (≤13.3% vs. >13.3%) (A), RDW (≤13.3% vs. >13.3%) and presence or absence of LGE (**B**) or the extent of the LGE (≤3.4% vs. >3.4%) (**C**). *RDW*, red cell distribution width; *LGE*, late gadolinium enhancement.

The Kaplan-Meier survival curves in the groups of patients categorized by the RDW and LGE-CMR characteristics (presence or absence and extent of the LGE) are shown in Fig. 2. Patients with >13.3% had a lower event-free survival rate than those with ≤13.3% (Log-rank, p < 0.0001) (Fig. 2A). In both patients with and without LGE, the possibility of experiencing a composite end point was significantly increased in patients with a higher RDW level than in those with a lower RDW level (Log-rank, p = 0.014 and p = 0.001, respectively) (Fig. 2B). Moreover, patients with a higher RDW level exhibited a significantly lower event-free survival rate than those with a lower RDW level in the subjects with a greater extent of the LGE (>3.4%) (Log-rank, p = 0.004), as well as in those with a smaller extent of the LGE (≤3.4%) (Log-rank, p = 0.005) (Fig. 2C). Patients with a higher RDW level and the presence or greater extent of the LGE had the lowest survival among the groups (overall Log-rank, p < 0.0001, respectively). On the other hand, the event-free survival rates of the higher RDW group with the absence or a smaller extent of the LGE were similar to those in the lower RDW group with the presence (Log-rank, p = 0.852) or greater extent (Log-rank, p = 0.925) of the LGE.

Table 3 demonstrates the Cox regression analyses for the primary endpoint of adverse events. A univariate Cox analysis showed significant associations between adverse events and the RDW (per 1% increase) (unadjusted hazard ratio [HR] 1.22, 95% confidence interval [CI] 1.11–1.33, p < 0.0001), BMI, diastolic BP, current smoker, hemoglobin, Ln PLT count, Ln NT-proBNP, eGFR_MDRD_ < ${60}_{(\mathrm{mL}/\min /1.73{{\rm{m}}}^{2})}$, use of digoxin, LVEDVI, presence and extent of the LGE, midwall and transmural distributions of the LGE. In multivariate analysis, the RDW (per 1% increase) (adjusted HR 1.17, 95% CI 1.04–1.33, p = 0.010) and presence (adjusted HR 3.41, 95% CI 1.82–6.41, p < 0.0001) and extent (per 1% increase) (adjusted HR 1.02, 95% CI 1.01–1.04, p = 0.010) of the LGE, along with an older age, lower diastolic BP, and being a current smoker were associated with a poor clinical outcome.

**Table 3:** Cox regression analyses for prediction of adverse clinical events.

Variables	Univariate analysis		Multivariate analysis	
	Unadjusted HR (95% CI)	p Value	Adjusted HR (95% CI)	p Value
RDW (%)	1.22 (1.11 – 1.33)	<0.0001	1.17 (1.04 – 1.33)	0.010
Age (years)	1.01 (0.99 – 1.02)	0.086	1.03 (1.01 – 1.04)	0.001
Male	1.07 (0.77 – 1.49)	0.671		
BMI (kg/m^2^)	0.96 (0.92 – 0.99)	0.038		
Diastolic BP (mmHg)	0.98 (0.97 – 0.99)	0.003	0.97 (0.96 – 0.99)	0.001
NYHA class	1.07 (0.86 – 1.32)	0.526		
Diabetes mellitus	1.23 (0.88 – 1.72)	0.220		
Hypertension	1.09 (0.79 – 1.50)	0.606		
Current smoker	1.45 (1.03 – 2.06)	0.035	1.74 (1.11–2.70)	0.015
Hemoglobin (g/dL)	0.85 (0.79–0.92)	<0.0001		
Ln PLT count	0.48 (0.31–0.73)	0.001		
Ln NT-proBNP	1.20 (1.05–1.38)	0.008		
eGFR_MDRD_ < 60 _(mL/min/1.73m_^2^_)_	1.73 (1.22–2.46)	0.002		
Use of digoxin	1.80 (1.31–2.48)	<0.0001		
LVEF (%)	0.98 (0.96–1.00)	0.072		
LVEDVI (mL)	1.01 (1.00–1.01)	<0.0001		
Presence of LGE	2.65 (1.74–4.05)	<0.0001	3.41 (1.82–6.41)	<0.0001
LGE extent (%)	1.03 (1.02–1.04)	<0.0001	1.02 (1.01–1.04)	0.010
Pattern of LGE				
No LGE	1 (reference)			
Subendocardial	1.51 (0.73–3.10)	0.268		
Midwall	2.04 (1.44–2.87)	<0.0001		
Subepicardial	1.70 (0.77–3.77)	0.190		
Transmural	2.86 (1.66–4.93)	<0.0001		
Patchy	1.12 (0.80–1.58)	0.501		

*RDW*, red cell distribution width; *BMI*, body mass index; *HR*, hazard ratio; *CI*, confidence interval; *BP*, blood pressure; *NYHA*, New York Heart Association; *Ln PLT*, log-transformed platelet; *Ln NT-proBNP*, log-transformed N-terminal pro-brain natriuretic peptide; *eGFR*_*MDRD*_, estimated glomerular filtration rate calculated using the Modification of Diet in Renal Disease formula; *LVEF*, left ventricular ejection fraction; *LVEDVI*, left ventricular end-diastolic volume index; *LGE*, late gadolinium enhancement.

**Figure 3:**
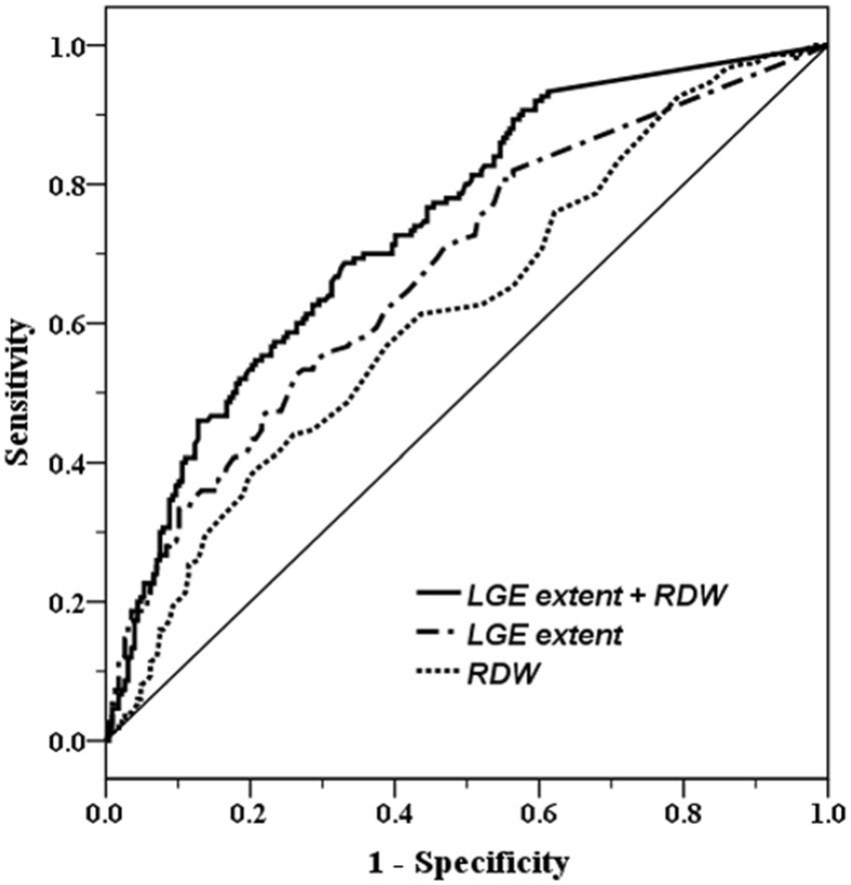
ROC curve analysis of RDW, LGE extent and the combination of RDW and LGE extent for predicting adverse clinical events. *ROC*, receiver operating characteristics; *RDW*, red cell distribution width; *LGE*, late gadolinium enhancement.

**Figure 4:**
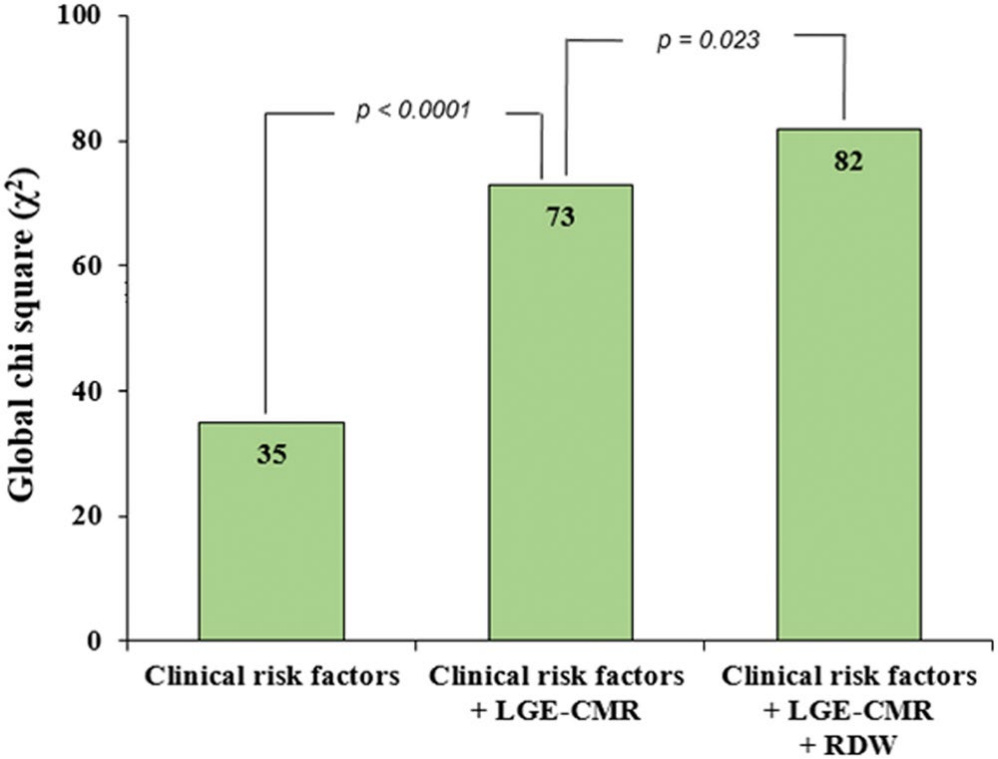
Incremental value of RDW for the prediction of adverse clinical events. Changes in the global χ^2^ were compared to assess the additive prognostic value of RDW when added to the prediction model of clinical risk factors and LGE-CMR data. *RDW*, red cell distribution width; *LGE*, late gadolinium enhancement; *CMR*, cardiac magnetic resonance. Clinical risk factors; *age, male, BMI, diastolic BP, NYHA class* ≥ *3, DM, HTN, current smoker, hemoglobin, Ln NT-proBNP, eGFR*_*MDRD*_ < 60. LGE-CMR data; *LVEF and presence, extent, and pattern of LGE*.

The receiver operating characteristics (ROC) analysis was performed to assess the combined value for RDW and LGE extent in predicting adverse clinical events. The area under the curve (AUC) for RDW, LGE extent and the combination of RDW and LGE extent were 0.618 (95% CI, 0.56– 0.68), 0.678 (95% CI, 0.62– 0.73) and 0.740 (95% CI, 0.69– 0.79), respectively (Fig. 3). Furthermore, the addition of the RDW into the model with the clinical risk factors and LGE-CMR data significantly improved the overall χ^2^ score over the model with the clinical risk factors plus LGE-CMR data alone (global χ^2^ 73 vs.82, p = 0.023) (Fig. 4).

## Discussion

The major findings of our study conducted in patients with NICM were: 1) RDW level was significantly higher in patients with LGE than in those without LGE, but it was not associated with the extent and distribution pattern of the LGE; 2) an elevated RDW predicted adverse clinical events independent of LGE-CMR characteristics, and it could provide an incremental predictive value for a poor outcome over a combination of the clinical risk factors and LGE-CMR. This was the first study to describe the relationship between the RDW and LGE-CMR, and to assess the additive prognostic value of the RDW over LGE-CMR data in patients with NICM. The clinical implication of RDW in patients with HF was first reported by Felker *et al*.^13^. In this large prospective study of 2,679 symptomatic chronic HF patients, higher RDW levels were associated with cardiovascular death or HF hospitalizations and all-cause mortality. The association between an elevated RDW and adverse outcomes was also found in patients with acute HF^14^, and the meta-analysis has shown a significant prognostic value of the RDW for HF patients^3^. However, most data were short-term follow-up studies including patients with HF of various etiologies. Recently, although Wasilewski *et al*. found a significant association between high RDW and long-term mortality in a subgroup of patients with non-ischemic LV systolic dysfunction, coronary angiogram to rule out ischemic etiology of HF with significant CAD was not performed in all participants^15^. The present study was also clinically meaningful in that it was the first study to evaluate the prognostic significance of RDW in a relatively large number of NICM patients who were completely confirmed by coronary angiogram or computed tomography.

HF is a complex clinical syndrome characterized by a reduced ability of the ventricle to fill or eject blood, which can be caused by many different conditions leading to any structural or functional cardiac abnormalities^16^. Interestingly, various factors causing hematopoietic dysfunction and anisocytosis such as inflammation, oxidative stress, nutritional deficiency, renal failure and aging also have been involved in the development or worsening of HF^2,5^. Özülkü *et al*. reported an independent negative association between the RDW level and coronary flow reserve in patients with idiopathic dilated CMP^17^. The authors described that higher RDW levels reflect a chronic inflammatory state, resulting in impaired coronary microcirculation and LV systolic dysfunction. In our study of NICM patients, there was a negative relationship between the RDW level and LVEF (r = –0.132, p = 0.017). Moreover, patients with LGE had a significantly higher level of the RDW, as well as higher levels of the CRP (3.3 ± 3.9 mg/dL vs. 2.1 ± 2.8 mg/dL, p = 0.008) and NT-proBNP (3245 [1393, 6523] pg/mL vs. 1769 [436, 4040] pg/mL, p < 0.0001) compared to those without LGE (Supplementary Figure S1). Given the pathogenesis of myocardial fibrosis following chronic inflammation, a high RDW seems to be associated with the inflammatory process of NICM^10^.

Several studies have shown a significant increase in the inflammatory markers and its relationship with a poor prognosis in NICM patients^18–21^. Vergaro G *et al*. reported a significant association between galectin-3 and myocardial fibrosis assessed by the LGE^20^. Furthermore, a recently published study demonstrated that growth differentiation factor-15, a cytokine involved in inflammation and fibrosis, was linked to an adverse cardiac remodeling and worsening functional capacity^21^. As mentioned above, the RDW may also be a strong surrogate marker of inflammation, which is one of the most important pathologic processes in HF. However, it still remains controversial whether an elevated RDW is just an epiphenomenon of an underlying biological or metabolic imbalance, or a real risk factor. Hemorheologic alterations of RBCs in conditions of high anisocytosis contribute to the inadequate myocardial perfusion, and the abnormal erythrocytes can directly behave as an active player in the pathogenesis of myocardial remodeling and fibrosis^22^. Allen LA *et al*. reported that not only inflammatory stress but also impaired iron mobilization could be a potential mechanism for the unfavorable prognostic effect of an elevated RDW^23^. In addition, an elevated RDW has been shown to have a significant association with poor CD34 (+) stem cell mobilization^24^, and some clinical trials have demonstrated the beneficial effects of stem cell therapy in NICM patients^25^. In our study, RDW was not correlated with the CRP level (p = 0.779), but it had a significant positive correlation with the NT-proBNP level (r = 0.383, p < 0.0001). Further, we found an independent association between an elevated RDW and an adverse outcome, and revealed an additive prognostic value of the RDW over the LGE-CMR characteristics. These findings suggested that the aforementioned hemorheological alterations, besides inflammation, may play an important role in the adverse prognostic impact of the RDW. Since the RDW reflects inflammation, as well as the hematopoietic activity affecting the progression of cardiovascular disease^2^, the RDW could be a reliable prognostic indicator in NICM patients.

Our study had several inherent limitations due to its retrospective design with a potential selection bias and limitations on the data acquisition. Although longitudinal RDW variations have been also associated with a poor prognosis and the association was even stronger than that for the baseline RDW^26–28^, a serial assessment of RDW was not available in our study. Moreover, we did not analyze the hemorheological parameters known to be well correlated with the RDW level, such as the RBC deformability or aggregation index and whole blood viscosity. Also, more sensitive and specific markers for inflammation than the CRP, and their relationships with the RDW were not investigated. Interestingly, our study population showed a significantly higher prevalence of LGE than those in a previously reported data^29^. Several studies have identified myocardial perfusion abnormalities and its association with myocardial fibrosis in patients with DCM, and described that microvascular dysfunction may play a role in the pathogenesis of DCM^30–32^. Microvascular dysfunction is well known to be associated with endothelial dysfunction linked to systemic inflammation^33^. Considering clinical characteristics of our study population having more atherosclerotic risk factors compared to prior studies, high prevalence of LGE in our study may suggest the presence of microvascular dysfunction. Unfortunately, we could not perform the additional functional studies to assess myocardial perfusion or coronary flow reserve due to retrospective design. In addition, our study population showed a high prevalence of patchy LGE, particularly at right ventricular insertion point (RVIP-LGE) that is generally considered to be nonspecific in patients with hypertrophic cardiomyopathy^34^. However, its clinical implication in other medical conditions still remains unclear, and in a recently published our study including 360 NICM patients, we found that patients with RVIP-LGE had a worse prognosis than those with absence of LGE^35^. Although tissue characteristics of RVIP-LGE was not confirmed by endomyocardial biopsy in this study, the image quality was checked, and the potential pitfalls and artifacts mimicking myocardial scar were also excluded by two experienced radiologists.

Finally, the identification and quantification of interstitial fibrosis using a novel imaging technique, such as T1 mapping or extracellular volume (ECV) estimates were not applicable and the serial change in the LGE-CMR characteristics during the follow-up was not assessed.

## Conclusion

Among patients with NICM, RDW level was significantly higher in subjects with LGE than in those without LGE. An elevated RDW was an independent predictor of adverse clinical outcomes, and we also found its additive prognostic value over LGE-CMR characteristics. The RDW could be an integrative prognostic marker representing both multiple pathologic factors and hemorheological alterations. A combined assessment of the RDW and LGE-CMR data may improve the risk stratification in NICM patients.

## Methods

### Study population

A total of 465 consecutive patients with newly diagnosed NICM who underwent CMR with gadolinium between May 2003 and February 2018 at two tertiary hospitals in South Korea were retrospectively enrolled. All patients underwent CMR just after the diagnosis of NICM based on a detailed history and clinical evaluation, including a 12-lead electrocardiogram (ECG), echocardiography, and coronary angiogram or coronary CT. NICM was defined in accordance with the World Health Organization/International Society and Federation of Cardiology’s guidelines as follows: (1) presence of symptoms or signs of HF according to the Framingham criteria; (2) a reduced LVEF (<50%) without regional wall motion abnormalities; (3) an increased LV end-diastolic dimension (LVEDD > 55 mm); (4) no prior history of a myocardial infraction or revascularization; and (5) absence of significant CAD on the coronary angiogram or coronary CT (>50% luminal narrowing of one or more coronary artery)^36^. Patients with medical conditions that could increase the plasma RDW levels, such as hematological diseases or a malignancy, chronic obstructive pulmonary disease, inflammatory bowel diseases, and severe arthritis were excluded. We also excluded subjects with acute myocarditis, significant valvular heart diseases (≥moderate degree), hypertrophic cardiomyopathy, and infiltrative cardiomyopathy or other specific cardiomyopathies, structural heart diseases, and prior cardiac surgery. Finally, 378 patients were identified as the study population. The study protocol conformed to the principles of the Declaration of Helsinki and was approved by the local ethics committee (EUMC 2017-09-019-004). The study protocol was approved by the Institutional Review Boards of two general hospitals, Ewha Womans University Mokdong Hospital and Severance Cardiovascular Hospital. All of the data were fully anonymized prior to access and informed consent was waived due to the retrospective nature of this study.

### Blood sampling

All laboratory data, including the routine blood chemistry and hematologic parameters such as the hemoglobin, hematocrit, mean corpuscular volume (MCV), and RDW, were obtained within 3 days of the CMR. The RDW was measured as part of the automated complete blood count using the Advia 2120i automated analyzer (Siemens Healthcare Diagnostics, Deerfield, IL, USA).

### CMR acquisition

All CMR imaging studies were performed on a 1.5-T scanner (Intera Achieva; Phillips Medical Systems, Best, The Netherlands or Phillips Healthcare, Andover, MA, USA) with a phase array cardiac coil at both centers. The CMR image acquisition has been reported previously^37^. ECG-gated cine images were acquired using a balanced steady-state free precession sequence with the following parameters: TR [repetition time]/TE [echo time], 3.4/1.7 ms; flip angle, 50°; field of view, 360 × 360 mm; matrix, 256 × 256 mm; slice thickness, 8 mm; 25 frames per cardiac cycle; average number of signals, 1; and short axis planes encompassing the entire LV without a slice gap. Delayed enhancement images were acquired 10 minutes after infusing intravenous gadolinium-DTPA (0.2 mmol/kg; gadoterate dimeglumine; Dotarem, Geurbet) at 2 mL/s using a T1-weighted 2-dimensional gradient echo inversion recovery sequence with the following scanning parameters: TR/TE, 5.3/1.6 ms; flip angle, 15°; field of view, 360 × 360 mm; matrix, 512 × 512 mm; slice thickness, 8 mm; average number of signals, 2; no interslice gap. The inversion time (T1) was individually optimized to nullify the signal of normal myocardium using a dedicated T1 determining sequence^38^.

### CMR data analysis

All CMR images were reviewed using a dedicated software program (CMR42, Circle Cardiovascular Imaging, Calgary, Alberta, Canada) by the consensus of two experienced radiologists blinded to the patient clinical data and outcomes^37^. The LGE was visually assessed using a modified 16-segment model of the LV^39^, and the patterns of LGE were classified either as subendocardial, midwall, subepicardial, transmural, or patchy (Supplementary Figure S2)^40^. The LGE volume was quantified from a short-axis stack of images using a full width at half maximum (FWHM) technique, and the regional of interest was drawn in the area of the maximum signal intensity of a visible LGE for the FWHM threshold. The myocardial and LGE volumes were estimated from the sum of each area for each slice, multiplied by the slice thickness. The extent of the LGE was expressed as a percentage of the LGE volume, which was calculated by dividing the LGE volume by the myocardial volume, with a quotient multiplied by 100. The LGE analysis was performed twice by two independent expert readers blinded to all the patient details^37^. The inter-observer agreement between the two readers with respect to the presence or absence of an LGE was substantial (kappa value = 0.827, p < 0.005). The intra-observer variability of the LGE quantification for the coefficient of variation (CV) and intra-class correlation coefficient (ICC) were 12.5% and 0.99 (95% confidence interval [CI] 0.97–0.99), respectively.

### Endpoints

After the baseline clinical and CMR data collection were completed, all medical records of all patients were reviewed in detail for the purposes of obtaining additional follow-up data. The primary endpoint was a composite of all-cause death, hospitalizations due to worsening HF and major arrhythmic events. We defined hospitalizations for HF as the first readmission with worsening signs or symptoms of HF requiring additional treatment for at least one of the following: (1) intravenous therapy (e.g. diuretics, vasodilators, and inotropes); (2) implementation of mechanical circulatory support or surgical intervention; and (3) use of ultrafiltration, hemofiltration, or dialysis. Major arrhythmic events included sustained ventricular tachycardia, ventricular fibrillation, appropriate implantable cardioverter defibrillator interventions, and sudden cardiac death (SCD). The cause and date of the death were confirmed using the information from the National Population Registry of Korea National Statistical Office, together with all available medical records at the time of the death. SCD was defined as an unexpected death from a cardiac cause occurring within 1 h of the symptom onset, or nocturnal death without any prior history of worsening symptoms.

### Statistical analysis

Continuous variables were presented as the mean ± standard deviation (SD) or median (interquartile range, [IQR]) and were compared using independent t-tests or the Mann-Whitney test. Categorical variables were described as the n (%) and were compared using χ^2^ or Fisher’s exact tests. The association between the RDW and characteristics of the LGE were analyzed using a Spearman’s correlation coefficient and Kruskal-Wallis H test, as appropriate. Differences in the adverse event-free survival curves between the RDW (≤or > the median of 13.3%) and LGE (presence or absence of an LGE and ≤ or > the median of the LGE extent, 3.4%) groups were compared using a log-rank test. To investigate the independent association between the RDW and composite end point, a multivariate Cox regression analysis with a forward selection method was performed using covariates identified as significant in the univariate analysis (p < 0.05), as well as established risk factors for adverse cardiac events, including the characteristics of the LGE-CMR. The HRs for the prediction of the end points with 95% CIs were calculated. A ROC curve analysis was performed to assess the combined predictive value of RDW and the extent of LGE. The incremental predictive value of the RDW for the composite end point was assessed in the three different predictive models by comparing the global χ^2^ score of each model using ANOVA F tests. The baseline model consisted of clinical risk factors (age, male, BMI, diastolic BP, New York Heart Association [NYHA] class ≥3, DM, HTN, current smoker, hemoglobin, Ln NT-proBNP, and eGFR_MDRD_ < 60). Additional models were compromised of the clinical risk factors plus the LGE-CMR data (LVEF and presence, extent, and pattern of LGE), or clinical risk factors and LGE-CMR data plus the RDW. All statistical analyses were conducted using software package SPSS 18.0 (SPSS Inc., Chicago, Illinois, USA) and *P* values <0.05 were considered statistically significant.

## Supplementary information

Supplementary figures.
