# Supplementary material for: Additive prognostic value of red cell distribution width over late gadolinium enhancement on CMR in patients with non-ischemic dilated cardiomyopathy
Source: Sci Rep. 2020 Jun 8;10:9212. doi: 10.1038/s41598-020-66198-0 (PMC7280504; doi:10.1038/s41598-020-66198-0)

Additive prognostic value of red cell distribution width over late gadolinium enhancement on CMR in patients with non-ischemic dilated cardiomyopathy

Jeong-Eun Yi[1], Hye-Jeong Lee[2], Young Jin Kim[2], Yookyung Kim[3], Boyoung Joung[4], Junbeom Park[5*]

[1]Department of Cardiology, Eunpyeong St. Mary's Hospital, The Catholic University of Korea, Seoul, Republic of Korea

[2]Department of Radiology, Research Institute of Radiological Science, The Yonsei University College of Medicine, Seoul, Republic of Korea

[3]Department of Radiology, Ewha Womans Mokdong's Hospital, Ewha Womans University College of Medicine, Seoul, Republic of Korea

[4]Yonsei University Health System, Yonsei Cardiovascular Hospital, Yonsei University College of Medicine, Seoul, Republic of Korea

[5]Department of Cardiology, Ewha Womans Mokdong's Hospital, Ewha Womans University College of Medicine, Seoul, Republic of Korea

Supplementary Figure S1 Comparisons of C-reactive protein (A) and NT-proBNP (B) levels between patients with and without LGE

NT-proBNP, N-terminal pro-brain natriuretic peptide; *LGE*, late gadolinium enhancement

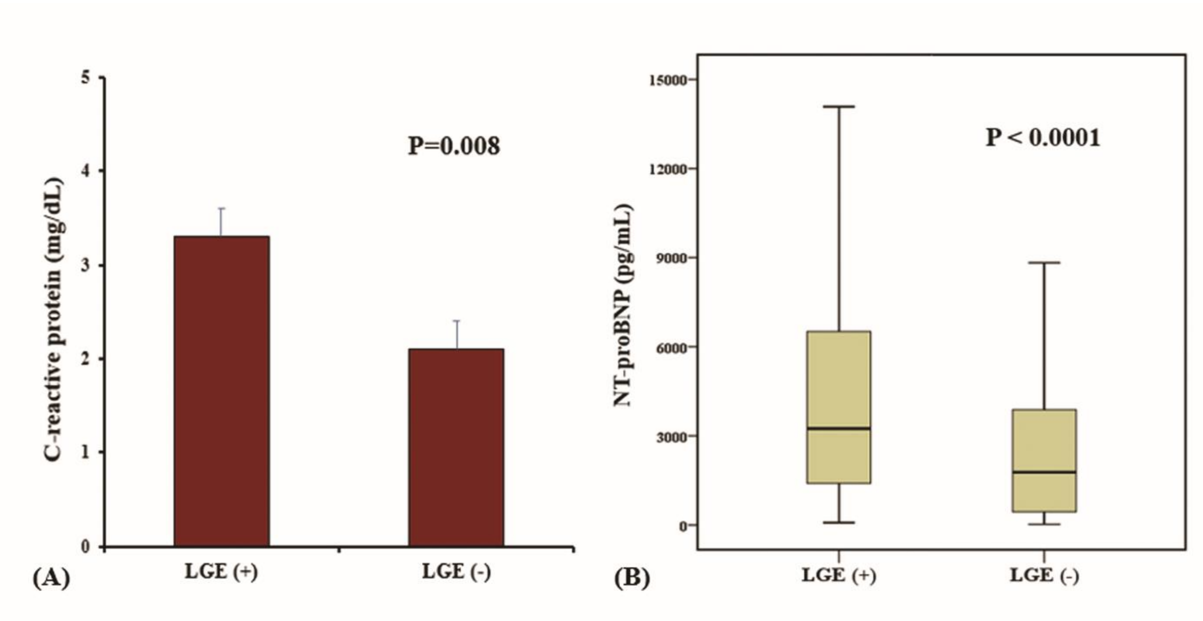

Supplementary Figure S2 Typical LGE patterns described among study population

LGE, late gadolinium enhancement

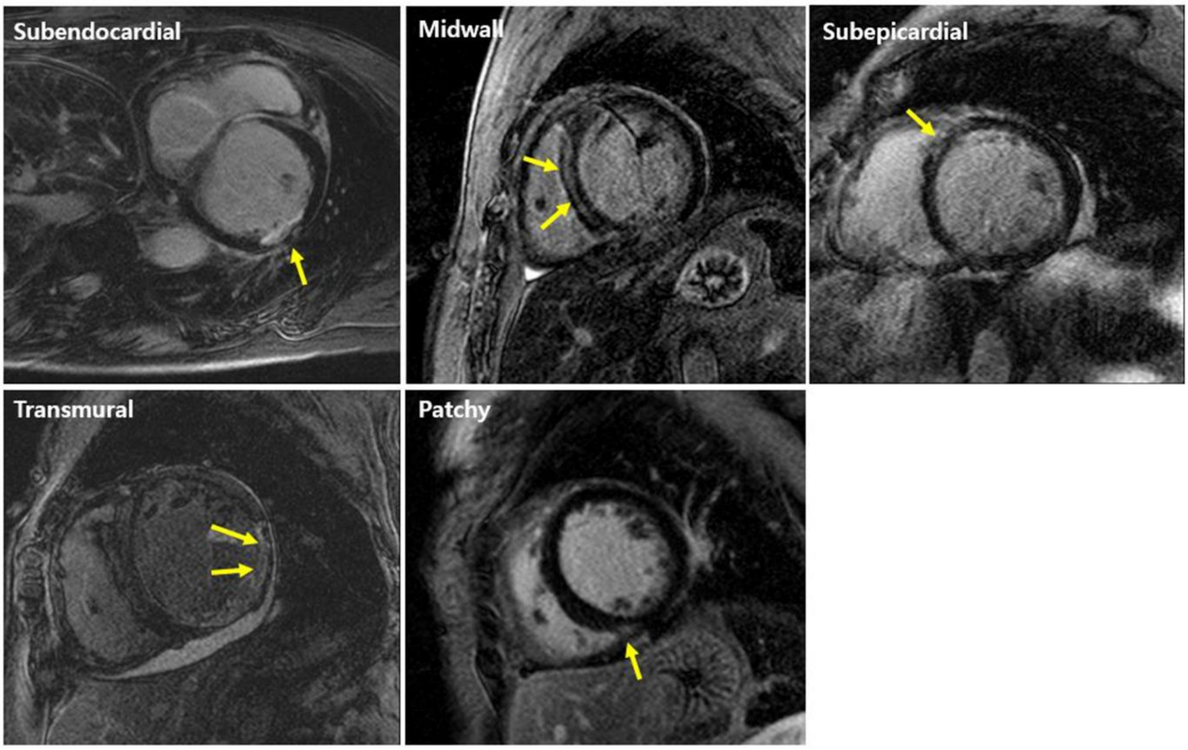

Supplement: Supplementary file 1 — Supplementary figures. [file 41598_2020_66198_MOESM1_ESM.pdf]
